# Clinical Lecture on Hernia*Delivered at the New York Post-Graduate Medical School and Hospital, March 28, 1895.

**Published:** 1895-07

**Authors:** W. B. De Garmo

**Affiliations:** Professor of Special Surgery


### CLINICAL LECTURE ON HERNIA.*

BY W. B. DE GARMO, M. D.

Professor of Special Surgery.

CASE I.—This man has been a patient at this clinic for eight years or more. The injection method was tried when he first came here and his return now shows the failure of that method. It was tried five or six times. The effect was, as it usually is, but temporary. He has delayed descent of the testicle on the same side. He cannot be cured now except by a cutting operation. The testicle did not come down until he was fifteen years of age. The hard rubber truss put on eight years ago is now in perfect condition, and does not show that it has been worn. The testicle is not larger than the ball of the thumb. In cases of this character, if the testicle is small and mixed up with a strangulated hernia, it is best to remove it. A partially developed testicle is often anchored in the canal and if it cannot be brought down it is best to take it out. This man could not, when we first saw him, be cured without danger. This was eight years ago. After treatment by injection, he went without a truss for one year. Heaton's fluid was used. Heaton's fluid is composed of fluid extract of white oak bark, rubbed up with solid extract of the bark and containing a little morphine. He has come to-day for a stronger spring, but the pressure of this one can be increased. The rubber on the inner side of the spring expands somewhat and therefore diminishes the pressure. I will heat and increase the curve and if it will not do, I will give him a stronger spring later.

The treatment of hernia by injection of white oak bark or other substances is very uncertain in its results and you cannot tell which cases are to be benefited. In view of its temporary action, and of the great advance both in safety and success by the present methods of operating, I have entirely abandoned the injections.

*Delivered at the New York Post-Graduate Medical School and Hospital, March 28, 1895.

This man has gradually acquired a bad habit that truss wearers are quite liable to, *i. e.*, of getting his truss too low, as you will see by the mark on the skin. The pad has been resting directly over the pubic bone, while the canal above has been wholly unprotected.

CASE II.—This twenty-four year old man was operated on three years ago and we found a peculiar state of affairs. He had had a hernia that was only partially reducible. I found omentum and a small cluster of cystic tumors like a bunch of white grapes, with thin sacs filled with fluid; it was united to the cord and hard to dissect off. I did Barker's operation, an operation that we do not do now. He has recurrence, as you see. There may be now a protrusion of omentum, or another growth of those cysts. The growth returned two months ago. The tumor is the size of a hen's egg and outside the external ring. It slips back on pressure, but even so may be cystic, and the case is liable to be a very deceptive one. I hold the mass against the pubic bone and can feel it slip back. The external ring is not large. There is little doubt that it is a piece of omentum. The hernia probably recurred because of adherence of the omentum at the internal ring, and the Barker operation did not open the canal, illustrating in this case a serious defect in the technique of that operation. By the method now used, the entire canal is exposed. I can give this man a choice between an operation, with far better prospect of permanent cure than he had before, and the wearing of a truss, which will probably have to be used for life. He has informed me that he prefers to have the operation.

CASE III.—This man is about fifty years old. He was here last week and has a double, incomplete, inguinal hernia, and a ventral hernia two and a half inches above the umbilicus. The contents of the ventral hernia are undoubtedly omentum. It is not larger than a pigeon's egg. It is in the median line, which is not usual, as they are more frequently found at the border of the rectus muscle. I have had a belt prepared for the ventral hernia. Such hernias rarely give trouble. This form of belt is similar to one used after operations for inguinal hernia. The incomplete, inguinal hernias require but little support. It is important that the belt should not be too tight, or the contents of the abdomen will be thrown farther down and interfere with the inguinal truss. Sometimes hernia is produced by action of a tight band of this character.

CASE IV.—Here is a boy aged eight years that has had a hernia for three years and will have it three years longer if he keeps on the same kind of truss that he is now wearing. The pad is on a descending arm that brings the pressure one and a half inches below the centre of the spring, and it gives no support over the internal ring. The canal is not closed at all.

The line between the tumor and the testicle is distinct, and this shows that the case is not of the congenital type. There is a very much thickened hernial sac. The canal is in bad condition. We have to deal with an acquired hernia with a very much thickened peritoneal covering. The external ring is much enlarged. There is about one chance to ten that the boy may be cured by mechanical means, wearing a truss three or four years. The hernia could be cured in two weeks by an operation. The two things to act against the cure by truss, are the thickened hernial sac, and the badly dilated canal. I think it is best to operate.* He would have to be brought once a month for a year or more if treated mechanically.

CASE V.—Here is a little girl, four and a half months old, and it is a question whether she has rupture or not. There is bulging on both sides of the abdomen, but this occurs when there is no hernia. The muscular walls may be weak. I do not find hernia but will take measure for a truss, as the mother gives a pretty clear history of its having been present. Little girls are easily cured by means of a truss, which must be worn not less than a year.

CASE VI.—This child, fourteen months old, was here last week. There is hernia on the right side, existing for the last three months. There is a protrusion into the upper part of the canal that can be discovered by sudden pressure over the internal ring, where the slipping of the hernial contents is felt. The hernia is acquired, and may be cured in one year, but must be seen once a month. The truss must not be taken off night or day, but raised up to wash under it when the child is quiet.

CASE VII.—Here is a woman with double hernia, following

*This boy was operated upon a few days later, and was entirely well at the expiration of ten days.

Alexander's operation. It has been a common experience to see hernias produced by this operation of shortening the round ligaments. I see fewer of these cases now than I did five years ago. Either the operation is not done so much, or the canal is closed better. I think it is due to the latter. The hernia has been as large as an egg, on the right side, smaller on the left. We will put on a truss. I have seen very few such cases get well by wearing a truss. These hernias came on thirteen months after the operation. It usually comes on the first year if at all. When the patient became pregnant it was first noticed in this case.

CASE VIII.—This man, thirty-six years old, suffered from hernia for fifteen years, and wore, for the most of that time, a German truss. Three years ago he found that the hernia could not be reduced. Since then it has never returned to the abdomen. It has given him almost constant trouble during the past year, and he has had occasional attacks of severe pain. Ten days ago, in your presence, I operated upon him, removing a mass of omentum as large as one's hand. To-day the bandages are removed for the first time, and, as you see, perfect primary union is found. The Bassini method for closing the canal was used. He has had no elevation of temperature or pulse, nor pain. He will leave the hospital at the end of two weeks from the date of the operation.

The operative treatment of these cases is successful to a degree that many of us never expected to attain.

Cases requiring the removal of omentum were formerly considered very dangerous, and showed a mortality rate of about fifteen per cent. But under the present improved method this is all changed. You have seen me operate on a number of these cases this winter, and in all, I have now operated upon over fifty of these omental hernias without a death or accident of any kind.

